# *Pinus koraiensis* essential oil enhances glucose uptake and proliferation in SH-SY5Y neuroblastoma cells

**DOI:** 10.1038/s41598-024-78357-8

**Published:** 2024-11-04

**Authors:** Hyungkuen Kim, Hwan Myung Lee, Sung-Jo Kim

**Affiliations:** Department of Biotechnology, College of Life and Health Sciences, Hoseo University, Baebang, Asan, 31499 South Korea

**Keywords:** *Pinus koraiensis*, Essential oil, Α-pinene, Glucose transporter, Neurodegenerative diseases, Cell death in the nervous system, Diseases of the nervous system

## Abstract

Aromatherapy using essential oils (EOs) is well known for its beneficial effects on mental health and neuroprotection. However, the significant molecular mechanisms have not yet been identified. Recent studies have identified a decrease in glucose uptake as a common feature across various neurodegenerative diseases (NDDs), leading to mitochondrial dysfunction and excessive autophagy. This suggests that glucose may serve not only as an energy source but also as a therapeutic target for NDDs. Using SH-SY5Y neuroblast-like cells and the glucose uptake inhibitor BAY-876, we demonstrated that glucose depletion promoted autophagy. To discover the potential therapeutics that modulate glucose metabolism, we obtained EO from *Pinus koraiensis* Siebold & Zucc. (PKSZ) using steam distillation. PKSZ-EO upregulated mRNA expression of *SLC2A2*, *SLC2A3*, and *SLC2A4*, leading to increased glucose uptake in SH-SY5Y cells. Furthermore, PKSZ-EO protected SH-SY5Y cells from BAY-876-induced mitochondrial dysfunction, cytostasis, autophagy, and inflammation. Using gas chromatography-mass spectrometry, we confirmed the high levels of α-pinene, an inducer of GLUT4 expression, in PKSZ-EO. These results suggest that PKSZ-EO exerts a protective effect against glucose depletion stress, highlighting its potential as a therapeutic agent for NDDs.

**Supplementary Information:**

The online version contains supplementary material available at 10.1038/s41598-024-78357-8.

## Introduction

Life is sustained by fundamental molecules such as oxygen, water, proteins, lipids, nucleic acids, and carbohydrates. Glucose, a simple carbohydrate, is paramount for energy metabolism and serves as a primary substrate for energy production and biosynthetic processes^1^. Dysregulated glucose homeostasis, including imbalanced glucose uptake, abnormal glucose transportation, decreased carbohydrate digestion, and impaired lysosomal glucose sensing, can lead to fatal diseases^2^. The brain, which regulates memory, learning, and motor control, consumes approximately 20% of the body’s metabolic energy due to its dense neuronal population. Neurodegeneration results in a diminished quality of life due to impairments in cognitive function. Aging and neurodegenerative diseases (NDDs), such as Alzheimer’s disease (AD), are associated with reduced glucose uptake^3,4^. Consequently, glucose regulation has emerged as a critical focus in neurodegeneration research.

Glucose uptake into cells is facilitated by glucose transporters (GLUTs), leading to their conversion to pyruvate, which is essential for mitochondrial energy production. In the brain, a variety of GLUTs are expressed, including GLUT1-6, and nerve cells use GLUT1-4 to take up glucose, with the downregulation of GLUT1/GLUT3 reported in AD neurons^5^. In SH-SY5Y neuroblast-like cells, which predominantly express GLUT1/GLUT2^6^, low-glucose conditions (0-0.25 mM) and the GLUT1 antagonist WZB117 induce cell death^7,8^. GLUT4 is important in the regulation of body glucose homeostasis, and when stimulated by insulin, leptin, or interleukin 6, it can translocate from the cytoplasm to the plasma membrane and upregulate glucose uptake in SH-SY5Y cells^9,10^. High-glucose conditions (100 mM) also induced cell death in SH-SY5Y cells^11^. Moreover, high-glucose conditions contribute to the synthesis of cholesteryl ester, a toxic intracellular accumulated form of cholesterol in the brain^12,13^. Therefore, we suggest that targeting glucose homeostasis is an effective strategy for the prevention and treatment of NDDs.

Autophagy, a key homeostatic process in the cellular digestion of glycogen, lipids, proteins, and organelles, can be induced by glucose depletion (GD) and infection^14^. The disruption of autophagy, marked by the accumulation of damaged mitochondria, is evident in non-infectious NDDs, such as multiple sclerosis, and is also a characteristic of neuropathic lysosomal storage diseases caused by mutations in lysosome-associated proteins^15,16^. This underscores the interconnectedness between nutritional status and autophagy in the prevention of neurodegeneration.

Pine trees, known for their diverse uses in furniture, therapeutic spaces, and aromatherapy, have been credited with mental health benefits supported by scientific findings that pine tree volatiles can lower cortisol levels^17,18^. Ethanol extracts of pine tree leaves have also been shown to protect against memory loss in a scopolamine-induced amnesia mouse model^19^. However, the therapeutic efficacy and molecular pathways of pine trees in NDDs remain unclear. In this study, we aimed to determine the effect of essential oils (EOs) from the nuts of *Pinus koraiensis* Siebold & Zucc. (PKSZ), commonly known as Korean pine, in GD-induced SH-SY5Y cells. We induced NDD-associated GD using BAY-876, a selective inhibitor of GLUT1, which is essential for transporting glucose from brain capillaries to neurons and is reduced in NDDs^20,21^. We evaluated whether PKSZ-EO affected glucose uptake in neurons by measuring GLUT expression levels, glucose uptake, and intracellular glycogen levels. We then identified substances in PKSZ-EO using gas chromatography-mass spectrometry (GC/MS). Overall, we validated the potential of PKSZ-EO to modulate glucose uptake and proliferation in SH-SY5Y cells.

## Results

### Cytotoxicity and antioxidant capacity of PKSZ-EO

**Fig. 1:**
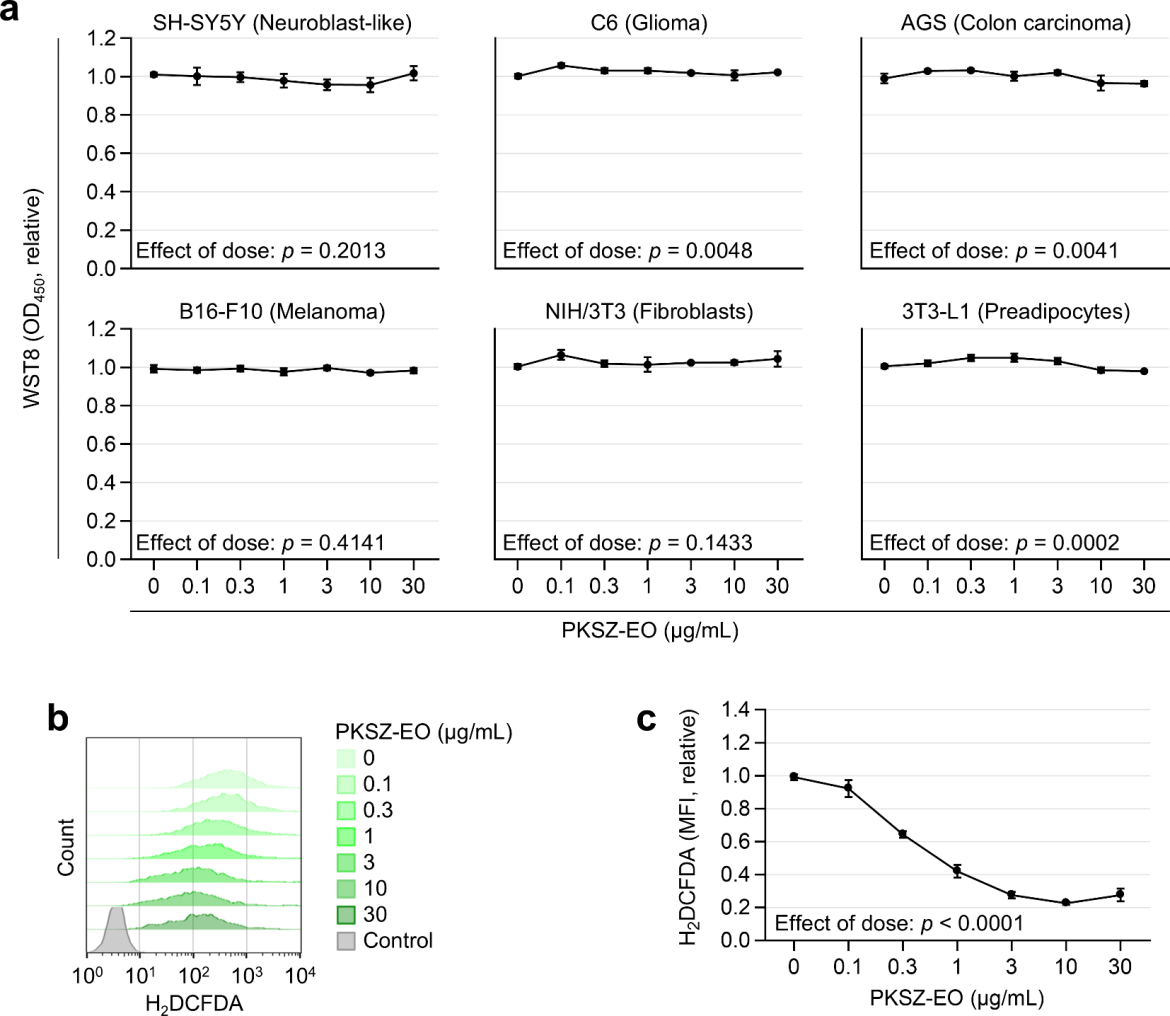
Cytotoxicity and antioxidant effect of PKSZ-EO in vitro. (**a**) Viability of SH-SY5Y, C6, AGS, B16-F10, NIH/3T3, and 3T3-L1 cells measured using WST8. Cells were cultured with PKSZ-EO for 48 h. (**b**,** c**) Intracellular reactive oxygen species levels measured using flow cytometry with H_2_DCFDA. SH-SY5Y cells were cultured with PKSZ-EO for 24 h. Representative flow cytometry profiles (**b**) and relative median fluorescence intensity (MFI) (**c**) are shown. p by one-way ANOVA (*n* = 3).

We first assessed the cytotoxicity of PKSZ-EO across various cell lines from brain-associated (SH-SY5Y, C6), colon (AGS), and skin (B16-F10, NIH/3T3, 3T3-L1) using the water-soluble tetrazolium salt-8 (WST8) viability assay. with PKSZ-EO treatment (0–30 µg/mL) showed no cytotoxicity in any of these cell lines (Fig. 1a). In SH-SY5Y cells, viability was slightly decreased at 10 µg/mL (not statistically significant). These results indicate that PKSZ-EO had no cytotoxicity up to a concentration of 30 µg/mL. Next, we confirmed the antioxidant capacity of PKSZ-EO in SH-SY5Y cells using flow cytometry with 2′,7′-dichlorofluorescein diacetate (H_2_DCFDA) (Fig. 1b,c). PKSZ-EO reduced intracellular reactive oxygen species levels (H_2_DCFDA fluorescence intensity) in a dose-dependent manner (0–10 µg/mL) in SH-SY5Y cells, suggesting a potential antioxidant effect of PKSZ-EO. Based on these results, a final concentration of 10 µg/mL PKSZ-EO was selected for further investigation.

### Protective effects of PKSZ-EO on SH-SY5Y cells against BAY-876-induced cytostasis

**Fig. 2:**
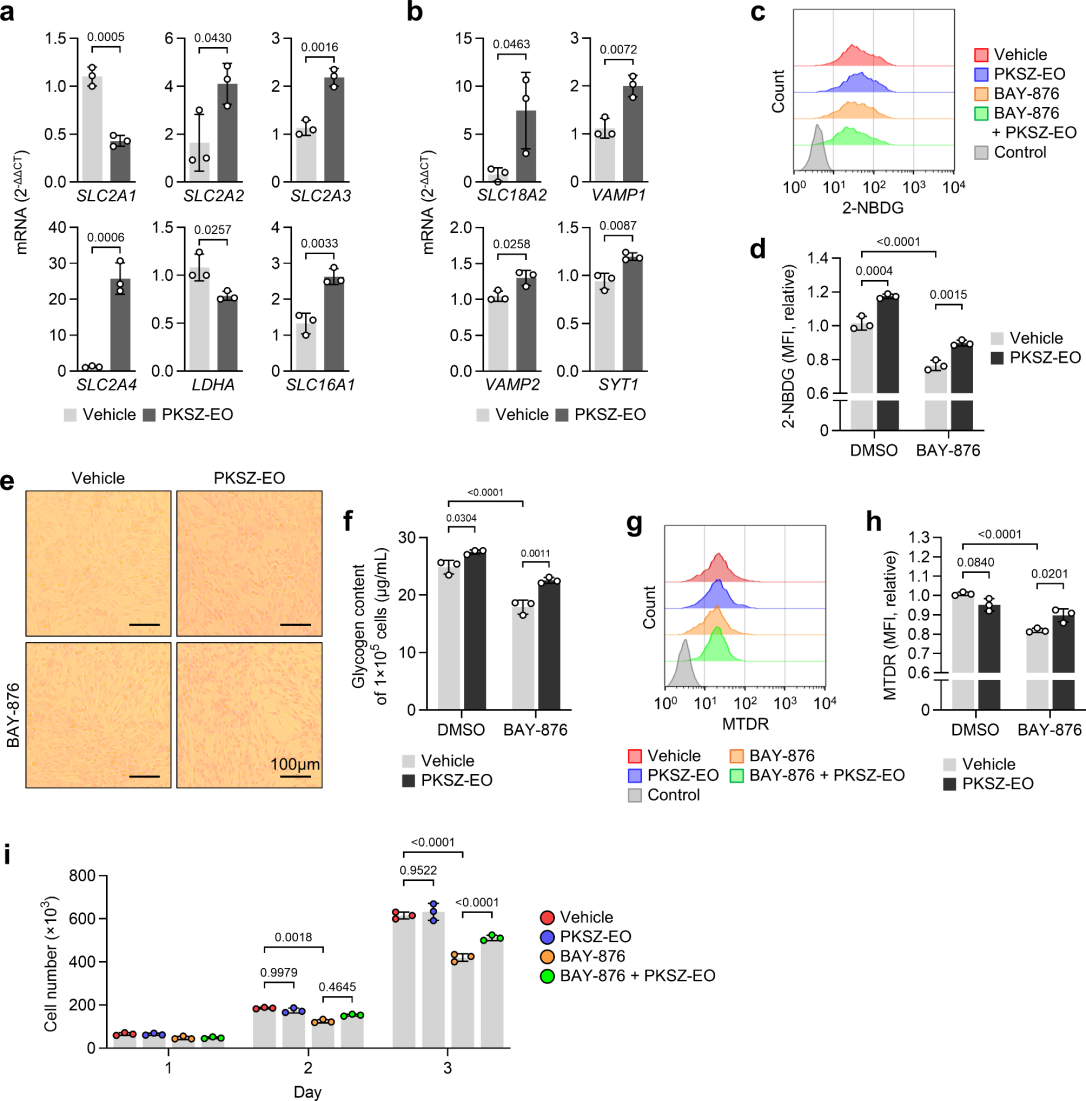
PKSZ-EO rescued SH-SY5Y cells from lowered glucose uptake by BAY-876. (**a**,** b**) mRNA expression levels of genes related to glucose metabolism (**a**) and SNARE (**b**) measured using qRT-PCR. SH-SY5Y cells were cultured with PKSZ-EO for 24 h. p by unpaired two-tailed Student’s* t*-test (*n* = 3). (**c**,** d**) Glucose (2-NBDG) uptake assay by flow cytometry in SH-SY5Y cells cultured with PKSZ-EO and/or BAY-876 for 24 h. Representative flow cytometry profiles (**c**) and relative MFI (**d**) are shown. (**e**) Best’s carmine glycogen staining in SH-SY5Y cells cultured with PKSZ-EO and/or BAY-876 for 48 h. (**f**) Quantification of the intracellular glycogen content using anthrone. SH-SY5Y cells were cultured with PKSZ-EO and/or BAY-876 for 48 h. (**g**,** h**) Flow cytometry of MTDR (mitochondrial activity) in SH-SY5Y cells cultured with PKSZ-EO and/or BAY-876 for 24 h. (**i**) Growth curve analysis of SH-SY5Y cells cultured with PKSZ-EO and/or BAY-876 for 72 h. p by two-way ANOVA with Tukey’s multiple comparison test (*n* = 3).

To confirm whether PKSZ-EO could upregulate glucose uptake, we measured the mRNA levels of GLUTs using quantitative real-time PCR (qRT-PCR). PKSZ-EO upregulated *SLC2A2*/*SLC2A3*/*SLC2A4* expression and decreased *SLC2A1* expression in SH-SY5Y cells. The expression of the lactate generator *LDHA* was decreased by PKSZ-EO in SH-SY5Y cells, and the expression of the lactate importer *SLC16A1* was increased (Fig. 2a). For docking of GLUT to the plasma membrane, soluble N-ethylmaleimide-sensitive-factor attachment protein receptor (SNARE) proteins are required^22^. In this study, the mRNA expression of SNARE-related genes, including *SLC18A2*, *VAMP1*, *VAMP2*, and *SYT1*, increased in SH-SY5Y cells following PKSZ-EO treatment (Fig. 2b). Increased glucose uptake by PKSZ-EO treatment in SH-SY5Y cells, both with and without BAY-876, was confirmed by flow cytometry with 2-NBD-glucose (2-NBDG) (Fig. 2c,d). Glycogen, a primary form of glucose storage in cells, was observed using Best’s carmine to accumulate in SH-SY5Y cells treated with PKSZ-EO (Fig. 2e). Moreover, the Antrone assay confirmed that PKSZ-EO upregulated the intracellular glycogen contents in BAY-876-treatedSH-SY5Y cells (Fig. 2f). These results indicate that increased glucose uptake due to the upregulation of *SLC2A2*/*SLC2A3*/*SLC2A4* could lead to pyruvate and acetyl-CoA generation rather than lactate accumulation.

Next, we assessed the mitochondrial activity and proliferation to confirm the protective effects of PKSZ-EO against GD-induced cytostasis. Flow cytometry with MitoTracker Deep Red (MTDR) confirmed that PKSZ-EO partially restored BAY-876-induced mitochondrial dysfunction in SH-SY5Y cells (Fig. 2g and h). Moreover, growth curve analysis showed that PKSZ-EO treatment protects the cytostasis caused by BAY-876 in SH-SY5Y cells (Fig. 2i; Table 1). These results suggested that PKSZ-EO protected SH-SY5Y cells from BAY-876-induced mitochondrial dysfunction and cytostasis by upregulating *SLC2A2*/*SLC2A3*/*SLC2A4* expression.

**Table 1:** Proliferation of SH-SY5Y cells. The increase in cell number and doubling time were calculated based on Fig. 2i.

	Vehicle	PKSZ-EO	BAY-876	BAY-876 + PKSZ-EO
Increased cell number (%, Day 3 vs. Day 1)	841.33 ± 24.49	882.38 ± 61.53	781.82 ± 36.6	958.62 ± 26.03
Doubling time (h, Day 3 vs. Day 1)	14.84	14.56	15.28	14.10

### PKSZ-EO reduces cholesteryl ester levels in SH-SY5Y cells

**Fig. 3:**
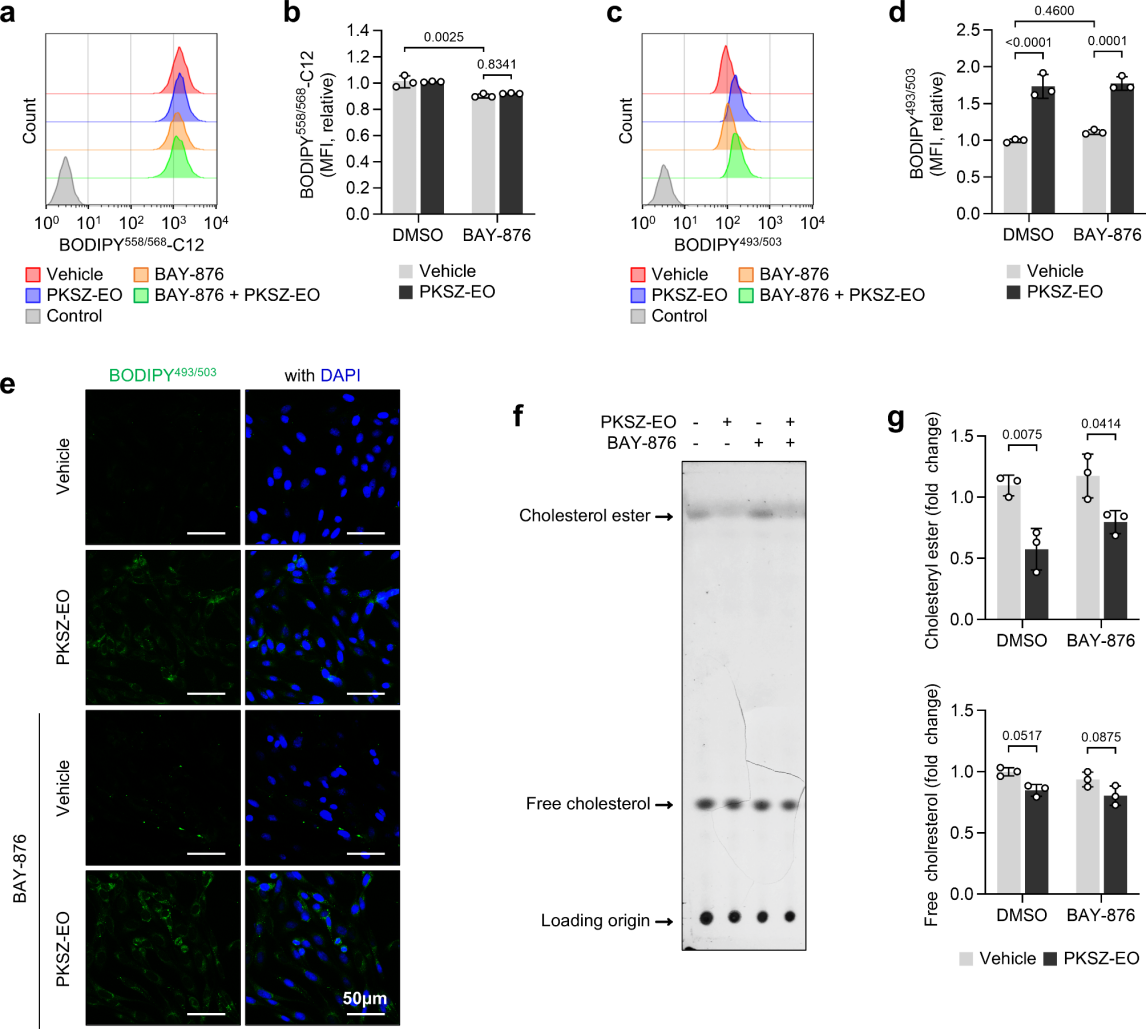
PKSZ-EO induced lipid accumulation in SH-SY5Y cells. (**a–g**) SH-SY5Y cells were cultured with PKSZ-EO and/or BAY-876 for 48 h (media/treatment were refreshed every 24 h). (**a**,** b**) Fatty acid (BODIPY^558/568^-C12) uptake assay using flow cytometry. (**c**,** d**) Intracellular neutral lipid levels measured using flow cytometry with BODIPY^493/503^. Representative flow cytometric profiles (**a**,** c**) and relative MFI (**b**,** d**) were shown. (**e**) Fluorescence microscopy of neutral lipids (BODIPY^493/503^) and nucleus (DPAI). (**f**,** g**) Quantification of intracellular cholesterol levels using TLC. (**f**) representative TLC image. (**g**) Quantification of lipid fold change. p by two-way ANOVA with Tukey’s multiple comparison test (*n* = 3).

Neutral lipids, known to be a storage form of lipids and glucose in the body, play a crucial role in brain structure and neuronal function, accounting for 60% of the dried weight of the brain. In addition, a recent study highlighted their significant role in regulating the differentiation and function of neural stem cells^23^. Therefore, we measured the neutral lipid levels to evaluate the effect of PKSZ-EO-induced glucose uptake on lipid metabolism. BAY-876 slightly reduced fatty acid (BODPY^558/568^-C12) uptake in SH-SY5Y cells, whereas PKSZ-EO did not induce changes in fatty acid uptake (Fig. 3a,b). Flow cytometry confirmed an increase in neutral lipid levels following PKSZ-EO treatment in SH-SY5Y cells, even when co-treated with BAY-876 (Fig. 3c,d), as observed by fluorescence microscopy (Fig. 3e). Next, we measured cholesterol levels using thin-layer chromatography (TLC) to evaluate the potential lipotoxicity of PKSZ-EO in SH-SY5Y cells. Unlike neutral lipid levels, PKSZ-EO downregulated cholesteryl ester levels, but no statistically significant changes in cholesterol levels were observed (Fig. 3f,g). These results suggested that PKSZ-EO-induced glucose uptake may lead to neutral lipid generation in SH-SY5Y cells and prevent cholesteryl ester accumulation.

### PKSZ-EO prevents BAY-876-induced autophagy and inflammation

**Fig. 4:**
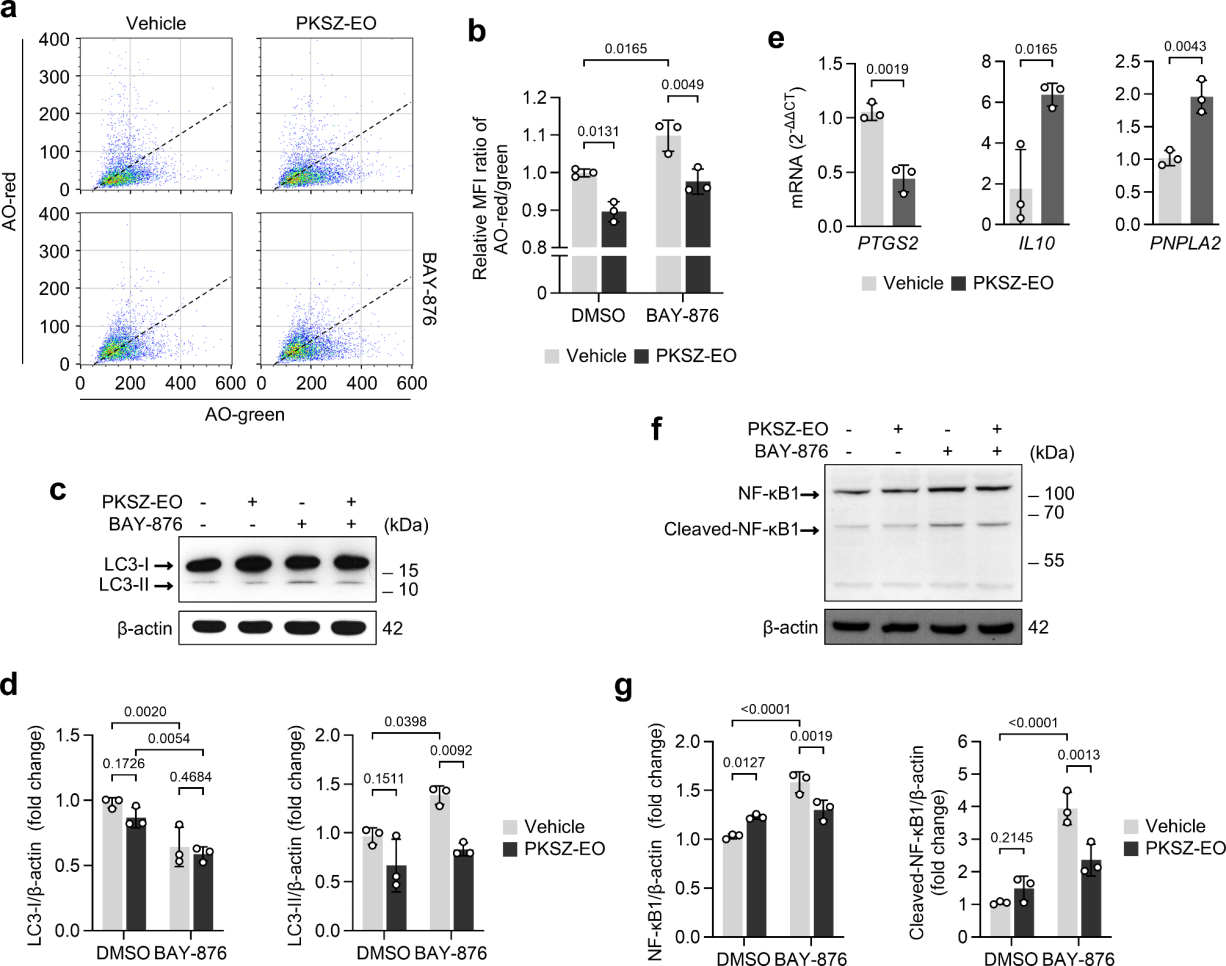
PKSZ-EO suppresses BAY-876-induced autophagy in SH-SY5Y cells. (**a**,** b**) Flow cytometry of autophagic activity (acridine orange; AO) in SH-SY5Y cells cultured with PKSZ-EO and/or BAY-876 for 48 h. (**b**) Relative MFI ratio of AO-red/green. (**c**,**d**) Immunoblotting of LC3 protein from SH-SY5Y cells cultured with PKSZ-EO and/or BAY-876 for 24 h. (**d**) Protein fold changes relative to β-actin. p by two-way ANOVA with Tukey’s multiple comparison test (*n* = 3). (**e**) mRNA expression levels of genes related to inflammation in SH-SY5Y cells cultured with PKSZ-EO for 24 h. p by unpaired two-tailed Student’s* t*-test (*n* = 3). (**f**,**g**) Immunoblotting of NF-κB1 protein from SH-SY5Y cells cultured with PKSZ-EO and/or BAY-876 for 24 h. (**g**) Protein fold changes relative to β-actin. p by two-way ANOVA with Tukey’s multiple comparison test (*n* = 3).

To investigate the influence of BAY-876-induced GD on autophagic stress, we quantified autophagic activity by flow cytometry with acridine orange (AO) and immunoblotting. We confirmed the upregulation of autophagic activity (red/green ratio of AO fluorescence intensity) in BAY-876-treated SH-SY5Y cells, which was downregulated by PKSZ-EO treatment (Fig. 4a,b). The conversion of LC3-I to LC3-II, a marker of autophagy, increased in BAY-876-treated SH-SY5Y cells and was prevented by PKSZ-EO treatment (Fig. 4c,d). These results suggest that PKSZ-EO suppresses GD (BAY-876)-induced autophagy in SH-SY5Y cells.

Next, we assessed the anti-inflammatory potential of PKSZ-EO in SH-SY5Y cells by qRT-PCR and immunoblotting. PKSZ-EO treatment suppressed the expression of the pro-inflammatory gene *PTGS2* and upregulated the expression of the anti-inflammatory genes *IL10* and *PNPLA2* (Fig. 4E). Adipose triglyceride lipase (encoded by *PNPLA2*), known as a trigger for neutral lipid degradation, helps maintain an anti-inflammatory phenotype in non-immune cells^24^. Cleavage (activation) of the pro-inflammatory transcription factor NF-κB1 was confirmed by immunoblotting. BAY-876 upregulated the expression and cleavage of NF-κB1 in SH-SY5Y cells, which was suppressed by PKSZ-EO (Fig. 4f and g). Taken together, these results suggest that PKSZ-EO exerts a protective effect against GD (BAY-876)-induced autophagy and inflammation in SH-SY5Y cells.

### Bioactive compounds in PKSZ-EO

**Fig. 5:**
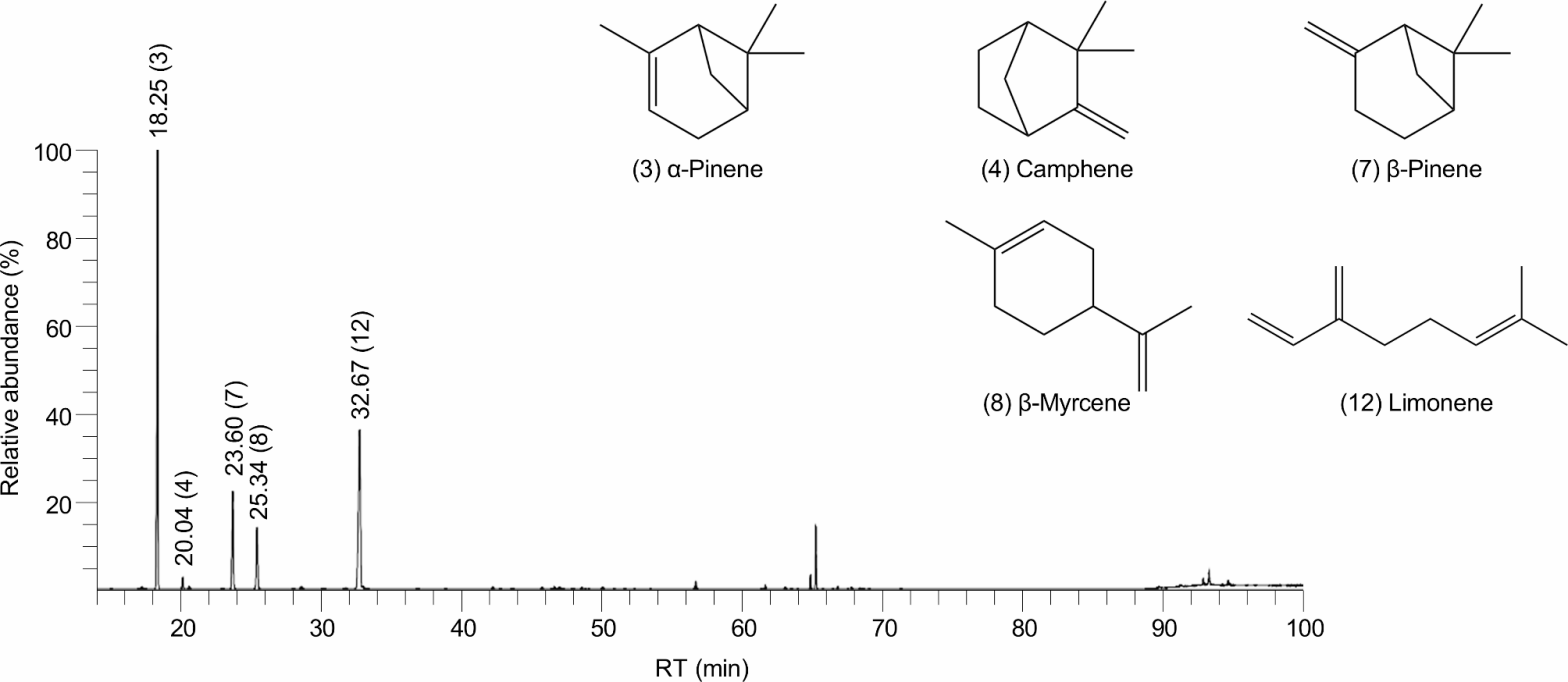
Identification of chemical composition in PKSZ-EO using GC/MS. Mass spectra with retention times (RT) and relative abundances are shown. The RT and chemical structures of the top 5 identified abundant compounds—α-pinene, camphene, β-pinene, β-myrcene, and limonene—are presented.

The chemical composition of PKSZ-EO was identified using GC/MS, and 30 compounds were identified (Fig. 5; Table 2). Five compounds were found to be abundant, each comprising over 1% of GC/MS area: α-pinene (46.57%), limonene (28.42%), β-pinene (12.02%), β-myrcene (8.09%), and camphene (1.22%). The high amount of terpenes, including α-pinene, in PKSZ-EO suggests its potential as a therapeutic agent for NDDs.

**Table 2:** Components of the *Pinus koraiensis* Siebold & Zucc. Essential oil determined using GC/MS. RT, retention time; RI, retention indices determined using a DB-5MS capillary column.

No.	Compound	RT	RI		Area (%)	CAS No.
			Observed	Literature		
1	Tricyclene	17.14	918	918	0.2	508-32-7
2	α-Thujene	17.41	920	920	0.06	353,313
3	α-Pinene	18.25	927	927	46.57	80-56-8
4	Camphene	20.04	942	942	1.22	79-92-5
5	Thuja-2,4(10)-diene	20.49	946	946	0.27	36262-09-6
6	Sabinene	22.9	966	966	0.12	3387-41-5
7	β-Pinene	23.59	972	972	12.02	127-91-3
8	β-Myrcene	25.34	986	986	8.09	123-35-3
9	3-Carene	28.5	1009	1009	0.33	13466-78-9
10	α-TERPINENE	30.07	1018	1018	0.14	99-86-5
11	p-Cymene	31.68	1028	1028	0.22	99-87-6
12	Limonene	32.67	1034	1034	28.42	138-86-3
13	β-Phellandrene	32.89	1035	1035	0.13	555-10-2
14	Eucalyptol	33.18	1037	1037	0.1	470-82-6
15	α-Terpinolene	42.15	1091	1091	0.19	586-62-9
16	α-Campholenal	45.65	1131	1130	0.12	4501-58-0
17	Limonene oxide, trans-	46.31	1141	1141	0.04	4959-35-7
18	Isopinocarveol	46.52	1144	1144	0.16	547-61-5
19	Verbenol	46.64	1146	1146	0.03	473-67-6
20	cis-Verbenol	46.87	1150	1152	0.12	1845-30-3
21	Camphor	46.97	1151	1151	0.08	76-22-2
22	Pinocarvone	47.91	1166	1164	0.04	30460-92-5
23	(E)-Pinanone	48.75	1179	1179	0.04	15358-88-0
24	Terpinen-4-ol	48.99	1182	1182	0.05	562-74-3
25	α-Terpineol	49.95	1197	1197	0.12	98-55-5
26	(1R)-(–)-Myrtenal	50.02	1198	1198	0.13	564-94-3
27	D-Verbenone	50.8	1210	1210	0.06	80-57-9
28	Trans-Carveol	51.55	1222	1222	0.05	1197-07-5
29	Carvone	53.41	1252	1252	0.05	99-49-0
30	Bornyl acetate	56.61	1303	1303	0.81	76-49-3
Total Identified (%)					100.00	

## Discussion

**Fig. 6:**
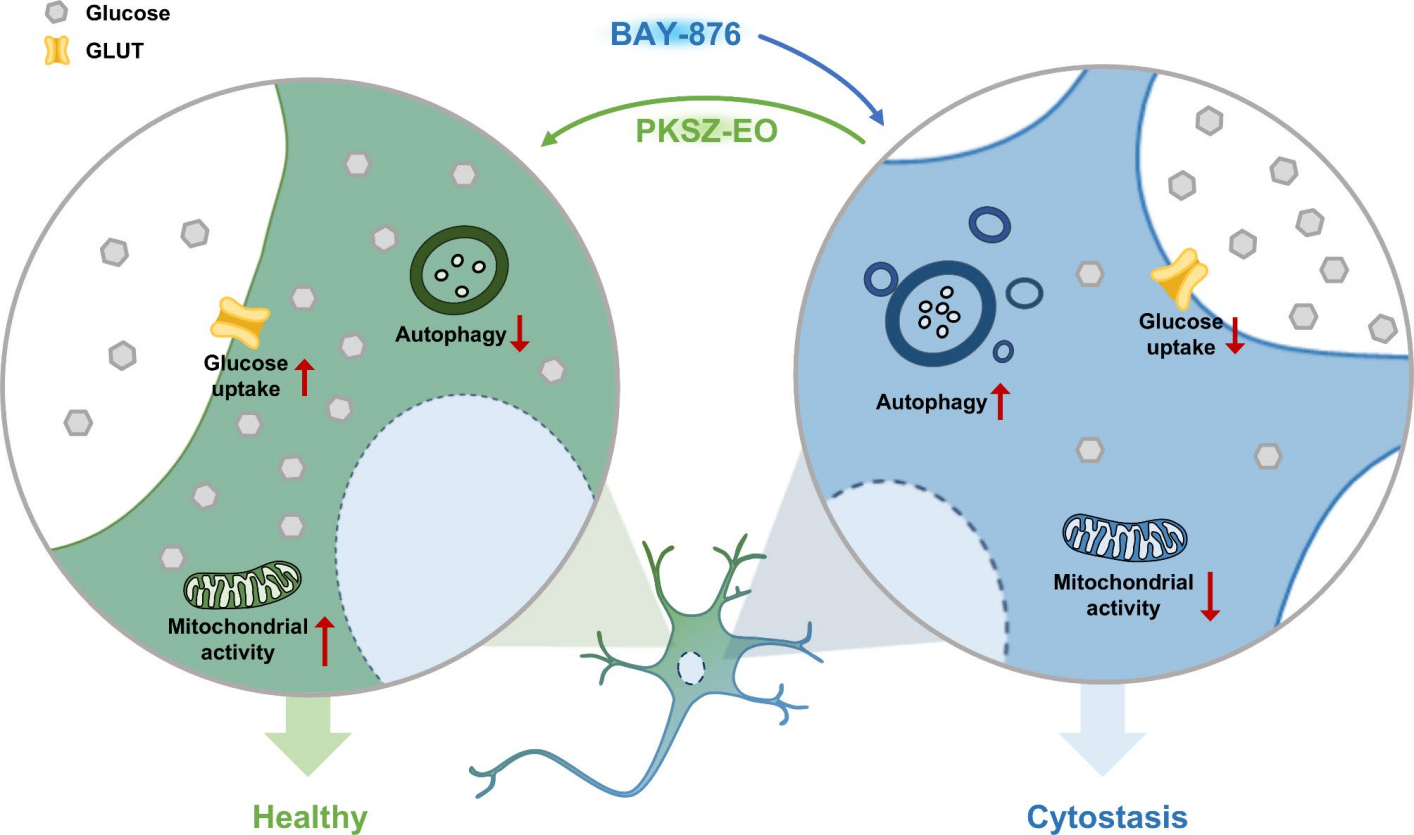
PKSZ-EO protected SH-SY5Y cells from BAY-876-induced cytostasis. In SH-SY5Y cells, BAY-876 treatment inhibited glucose uptake, leading to increased autophagy, defective mitochondrial activity, and cytostasis. PKSZ-EO treatment protected SH-SY5Y cells from BAY-876-induced cytostasis by upregulating glucose uptake.

Dysregulated glucose homeostasis is crucial for NDD pathogenesis; however, the mechanisms and treatments remain unclear. This study showed that BAY-876 induced GD-associated pathogenesis in SH-SY5Y cells, including reduced mitochondrial activity and proliferation and increased autophagy and inflammation. These effects were reversed by PKSZ-EO treatment, suggesting its potential as a therapeutic agent for GD-associated NDDs (Fig. 6). In addition, PKSZ-EO exhibited no cytotoxicity in skin cells, glial cells, and digestive organ cells, suggesting a low risk of side effects such as skin irritation, a well-known side effect of EOs.

Blood-brain barrier (BBB) permeability is essential for NDD therapeutics, and the terpenes in EOs exhibit this property^25^. PKSZ-EO contained terpenes, including α-pinene, β-pinene, and limonene. α-Pinene enhances GLUT4 expression and anti-inflammatory effects in mice^26^. Moreover, β-pinene reduces plasma glucose and lipoprotein levels in diabetic mice^27^, and limonene promotes glucose uptake and lipid accumulation in 3T3-L1 cells^28^. In this study, PKSZ-EO treatment in SH-SY5Y cells enhanced *SLC2A4* expression, reduced cholesteryl ester levels, and increased neutral lipid accumulation. GC/MS confirmed high levels of α-pinene (46.57%), β-pinene (12.02%), and limonene (28.42%) in PKSZ-EO. Notably, PKSZ-EO had a higher α-pinene content than the previously reported amounts of α-pinene (24%), β-pinene (12%), and limonene (28%) in *Pinus koraiensis* EO^29^. Overall, these findings suggest the potential therapeutic efficacy of PKSZ-EO in NDD, based on the regulation of GLUT expression by BBB-permeable terpene constituents.

GLUT1 is the only GLUT involved in the transporting glucose from brain capillaries, through endothelial cells, astrocytes, and into neurons. And GLUT1 is commonly downregulated in NDDs^20,21^. Therefore, we used BAY-876, a GLUT1 specific antagonist to mimic GD-associated NDD in vitro. Here, PKSZ-EO treatment specifically upregulated *SLC2A2*/*SLC2A3*/*SLC2A4* expression but not *SLC2A1* expression. GLUT3 has a higher transport capacity for glucose than other GLUTs and is highly expressed in brain areas with high neural activity. GLUT3 levels also decline with aging, and restoring its function is a potential target for anti-aging therapies^30^. GLUT4 is found at high levels in the hippocampus and is closely associated with the development of hippocampal insulin resistance-related factor disorders observed in AD. Insulin treatment is effective in improving cognitive function in AD patients, and GLUT4 blockers can impair hippocampus-dependent retrospective memory^31^. Overall, we suggest that PKSZ-EO likely activates neuronal metabolism not by improving glucose supply from the blood due to abnormalities in GLUT1, but by increasing glucose uptake in neurons by GLUT3/GLUT4.

We observed that BAY-876-treated SH-SY5Y cells exhibited reduced glucose uptake, mitochondrial activity, and proliferation. The accumulation of amyloid β peptide (Aβ), the most characteristic feature of AD, may interfere with the SNARE-mediated docking of GLUT3 and GLUT4 to the plasma membrane, resulting in reduced glucose uptake in neurons^22,32,33^. In our study, PKSZ-EO upregulated *SLC2A3*/*SLC2A4* and SNARE complex genes in SH-SY5Y cells, suggesting that PKSZ-EO may synergistically upregulate glucose uptake through GLUT expression and plasma membrane docking. Lactate is a glycolytic pathway metabolite that has been suggested to contribute to ion homeostasis and synaptic signaling. MCT1 facilitates lactate transport from astrocytes or oligodendrocytes to neurons and is decreased in AD model mice brain^34,35^. PKSZ-EO treatment induced *SLC16A1* (encoding MCT1) expression in SH-SY5Y cells, suggesting that PKSZ-EO influences both glucose and lactate homeostasis in neurons. From our results, we suggest that PKSZ-EO has therapeutic potential in managing GD in neurons by regulating glucose uptake and glycolysis.

PKSZ-EO induces neutral lipid accumulation in SH-SY5Y cells. Neurons maintain low neutral lipid levels and have a limited capacity for fatty acid catabolism^36^. Consistent with this, we observed only small amounts of neutral lipids in SH-SY5Y cells using fluorescence microscopy. Under healthy conditions, fatty acids in neurons can be expelled and taken up by neighboring astrocytes and microglia^36^. Since we cultured only neuroblast-like SH-SY5Y cells, astrocyte- or microglia-mediated fatty acid transport could not occur. In addition, PKSZ-EO-induced lipid accumulation was not altered by BAY-876 treatment, indicating that lipid accumulation is not solely due to PKSZ-EO-induced glucose uptake. Thus, although we cannot conclusively determine the lipogenic pathway of PKSZ-EO, lipid accumulation in SH-SY5Y cells may be a complex result of reduced autophagy and increased glucose uptake following PKSZ-EO treatment.

We confirmed that BAY-876 induces GD and cytostasis by increasing autophagic activity. PKSZ-EO treatment reduced autophagic activity and LC3-II levels. Consistent with other studies, GD can prompt autophagy to supply energy to cells, and uncontrolled excessive autophagy can lead to autophagic cell death, even after reintroducing glucose^37^. Thus, inhibition of autophagy is crucial for rescuing neuronal survival. PKSZ-EO not only upregulated glucose uptake but also inhibited autophagy, thereby improving cell proliferation. Therefore, the therapeutic application of PKSZ-EO may be suitable for treating NDDs with excessive autophagy.

Generally, a positive association exists between glucose levels and NF-kB activation, with hyperglycemia-induced inflammation. In this study, PKSZ-EO increased glucose levels, downregulated *PTGS2* expression and upregulated *IL10* and *PNPLA2* expression. Immunoblotting confirmed that PKSZ-EO treatment increased NF-κB1 levels in SH-SY5Y cells. Thus, these anti-inflammatory features of PKSZ-EO may be attributed to its antioxidant effect and weak association with glucose uptake in SH-SY5Y cells. In this study, BAY-876 exposure increased NF-κB1 and cleaved NF-κB1 levels in SH-SY5Y cells. In addition to regulating cell survival, apoptosis, and initiating inflammation, NF-kB is involved in the control of autophagy. IKK/NF-κB signaling upregulates autophagy via LC3 expression^38^. We observed that BAY-876 induced NF-κB1 expression was accompanied by autophagy and LC3 expression, indicating that increased NF-κB1 levels may have resulted from GD (BAY-876)-induced autophagy in SH-SY5Y cells.

However, this study had several limitations. As PKSZ-EO selectively induced the expression of *SLC2A2*/*SLC2A3*/*SLC2A4*, it is necessary to validate the neuroprotective efficacy of PKSZ-EO in situations where GLUT3/GLUT4 is inhibited. Since we did not observe direct evidence of BAY-876-induced cell death, validation of the protective efficacy of PKSZ-EO against GD-induced neuronal death is needed. As glucose uptake depends not only on GLUT expression, but also on the plasma membrane docking of GLUTs, it is necessary to analyze the cellular localization of GLUTs. Further experiments with in vivo NDD models or in vitro NDD organoids are required to confirm the therapeutic effect of PKSZ-EO in NDDs.

## Conclusion

In conclusion, we demonstrated that PKSZ-EO rescued the metabolic phenotypes of BAY-876-treated SH-SY5Y cells, which was supported by an increase in *SLC2A2*/*SLC2A3*/*SLC2A4* expression, glucose uptake, and proliferation. Given the importance of glucose metabolism in neurodegeneration, future studies will be interesting to determine the effects of PKSZ-EO on NDDs. Our study not only contributes to the understanding of the molecular mechanisms underlying the neuroprotective properties of PKSZ-EO but also opens new avenues for the development of therapeutics targeting glucose homeostasis in NDDs.

## Methods

### Preparation of *Pinus koraiensis* Siebold & Zucc. essential oil

Preparation of PKSZ nuts and extraction of essential oils were performed as described previously, with minor modifications^39^. Briefly, nuts of PKSZ were steam-distilled at 100 °C and then cooled to 4 °C. Afterward, the steam-distilled aqueous layer and EO layer were separated and stored at -80 ℃. The EO layer was diluted in dimethyl sulfoxide (DMSO) to a concentration of 10 mg/mL.

### Cell culture and experimental conditions

SH-SY5Y (CRL-2246), C6 (CCL-107), AGS (CRL-1739), B16-F10 (CRL-6475), NIH/3T3 (CRL-1658), and 3T3-L1 (CL-173) cells were purchased from American Type Culture Collection (Manassas, VA, USA). Cells were cultured in Dulbecco’s modified Eagle’s medium (10-013-CVR, Corning, Corning, NY, USA) supplemented with 10% (v/v) fetal bovine serum (TMS-013-BKR, Merck Millipore, Burlington, MA, USA) and 1% (v/v) penicillin-streptomycin (LS202-02, Welgene, Gyeongsan-si, Gyeongsangbuk-do, Korea) in a 5% CO_2_ atmosphere at 36.5 °C.

Cells were seeded in suitable cell culture ware and incubated for 12 h, then treated with PKSZ-EO, BAY-876, or vehicle (0.1% (v/v) DMSO), incubated as described in the respective figure legends, and subjected to in vitro assays. Unless otherwise indicated, PKSZ-EO was treated at a final concentration of 10 µg/mL, and BAY-876 at a final concentration of 1 nM. The treatment medium was replaced every 24 h.

### In vitro cytotoxicity and proliferation assay

To quantify cytotoxicity (cell viability), 2 × 10^3^ cells were seeded onto a 96-well plate, treated with PKSZ-EO for 48 h, and cultured with 10% (v/v) WST8 reagent (QM2500, BIOMAX, Seoul, Korea) for 2 h. The optical density (OD) at 450 nm was measured using FilterMax F3 (Molecular Devices, San Jose, CA, USA).

Proliferation was measured using a growth curve analysis. Cells (2 × 10^3^) were seeded onto 12-well plates and treated as indicated. To quantify the number of viable cells, the cells were harvested and stained with trypan blue solution (15250-061, Thermo Fisher Scientific, Waltham, MA, USA) for 1 min. Cell numbers were counted using a hemocytometer. Doubling times were calculated using Nonlinear Regression with an Exponential Growth Equation in GraphPad PRISM software ver. 10.1.0 (GraphPad Software, San Diego, CA, USA).

### Flow cytometry

Cells were prepared in complete media, cultured for 30 min with fluoroprobes, washed with phosphate-buffered saline (PBS; pH 7.4), and an average of 3 × 10^3^ cells was used for flow cytometry. A Guava EasyCyte cytometer (Merck Millipore) and Guava InCyte software ver. 2.6 (Merck Millipore) was used for flow cytometry. Flow cytometry profiles and fluorescence intensities were obtained using FlowJo ver. 10.6.2 (TreeStar, Ashland, OR, USA). Final concentration at 1 µM of H_2_DCFDA (35845, Sigma-Aldrich, St. Louis, MO, USA), 50 µM 2-NBDG (11046-10MG, Cayman Chemical, Ann Arbor, MI, USA), 50 nM MTDR (M22426, Invitrogen, Carlsbad, CA, USA), 10 µM BODIPY^558/568^-C12 (D3835, Invitrogen), 1 µM BODPIY^493/503^ (D3922, Invitrogen), and 1 µg/mL AO (A6014, Sigma-Aldrich) were used in this study. For the glucose and fatty acid uptake assays, cells were prepared in PBS containing 0.1% (w/v) bovine serum albumin.

### Quantitative real-time PCR

RNA isolation, cDNA synthesis, and qRT-PCR were performed according to the manufacturer’s instructions. Total RNA was isolated using Trizol (15596026, Invitrogen), and cDNA was synthesized from 1 µg RNA using WizScript cDNA Synthesis Kit (W2202, Wizbiosolutions, Seongnam-si, Gyeonggi-do, Korea) with oligo(dT) primers. qRT-PCR was performed with 10 ng cDNA and SYBR Green qPCR Master Mix (DQ485, BioFACT, Daejeon, Korea). StepOnePlus RT-PCR System (Applied Biosystems, Foster City, CA, USA) and StepOne software ver. 2.3 (Applied Biosystems) were used for qRT-PCR. mRNA fold changes were normalized to *ribosomal protein L13a* (*RPL13A*) using the 2^(−ΔΔCt)^ method. The primers used in this study are listed in Table 3. The raw data from the qRT-PCR is shown in Table S1.

**Table 3:** Primer sequences used in this study. The target gene, sequence, and GenBank accession numbers of the primers used for qRT-PCR are shown.

Gene	Primer sequence (5′ to 3′)		Accession No.
	Forward	Reverse	
*RPL13A*	CTCAAGGTGTTTGACGGCATCC	TACTTCCAGCCAACCTCGTGAG	NM_001270491.1
*SLC2A1*	GTATCGTCAACACGGCCTTC	GGAACAGCTCCTCGGGTGTC	NM_006516.4
*SLC2A3*	GTGGAGAAGGCAGGGCGACG	CAGTCTCTGTAGCTCCTAGG	NM_000340
*SLC2A3*	GAGATGAAAGATGAGAGTGC	CGATGCTGTTCATCTCCATG	NM_006931.3
*SLC2A4*	CAGCCATGAGCTACGTCTCC	GGCCCTAAATACTCAAGTTC	NM_001042.3
*LDHA*	GGATCTCCAACATGGCAGCCTT	AGACGGCTTTCTCCCTCTTGCT	NM_005566
*SLC16A1*	TTGTTGGTGGCTGCTTGTCAGG	TCATGGTCAGAGCTGGATTCAAG	NM_003051
*SLC18A2*	GCTATGCCTTCCTGCTGATTGC	CCAAGGCGATTCCCATGACGTT	SLC18A2
*VAMP1*	GACCAGTAACAGACGACTACAGC	TGCAAGGCATCAGCTCGGTCAT	NM_014231
*VAMP2*	CTCCAAACCTCACCAGTAACAGG	AGCTCCGACAGCTTCTGGTCTC	NM_014232
*SYT1*	GCTGACTGTTGTCATTCTGGAGG	CTTCAGCCTCTTACCATTCTGCA	NM_005639
*PTGS2*	CGGTGAAACTCTGGCTAGACAG	GCAAACCGTAGATGCTCAGGGA	NM_000963
*IL10*	CTGAGAACAGCTGCACCCAC	CATTCTTCACCTGCTCCACG	GQ405199.1
*PNPLA2*	CCCACTTCAACTCCAAGGACGA	GCAGGTTGTCTGAAATGCCACC	NM_020376

### Best’s carmine glycogen staining

Best’s carmine staining was performed as described by^40^ with minor modifications. Briefly, Cells were washed twice with PBS, fixed for 15 min with 3.8% (w/v) formaldehyde (F8775-500ML, Sigma-Aldrich), then washed with PBS. Then, washed with dH_2_O for 5 min, stained with 2.5% (w/v) Best’s Carmine solution (C1022, Sigma-Aldrich) for 30 min, washed with differentiating solutions containing 2% (v/v) methanol and 4% (v/v) ethanol in dH_2_O for twice. Washed for 1 min with ethanol (80%, 96%, and 100%), captured the images using Leica (Deerfield, IL, USA) DMi8 fluorescence microscope and LAS X software ver. 2.0.0.14332.

### Quantification of intracellular glycogen content

The intracellular glycogen content was measured as described previously^41^. Briefly, 1 × 10^5^ cells were suspended in 50 µL 30% (w/v) KOH and lysed for 20 min at 100 °C. Ethanol (150 µL) and dH_2_O (200 µL) were then added to the cell lysates. The cell lysate (50 µL) was reacted with 100 µL of 0.2% (w/v) anthrone (319899, Sigma-Aldrich) for 20 min at 100 °C. The OD_620_ of the cell lysate and glucose standards was measured using a FilterMaxF3 microplate reader.

### Neutral lipid staining

Cells were fixed in 3.8% formaldehyde for 15 min, washed with PBS, stained with 1 µM BODIPY^493/503^ for 30 min, washed with PBS, stained with diamidino-2-phenylindole (DAPI; D8417-10MG, Sigma-Aldrich), and imaged using Leica DMi8 fluorescence microscope.

### Lipid isolation and TLC

Lipid isolation and TLC were performed as previously described^42^. Briefly, Lipids from the 5 × 10^6^ cells were spotted on a silica plate (P46021, Analtech, Newark, DE, USA) and developed with a solvent containing hexane/ethyl ether/acetate = 80/20/2 (v/v/v). Lipids were visualized by incubation with 10% (v/v) sulfuric acid for 1 h at 120 ℃ and quantified using densitometry.

### Immunoblotting

Immunoblotting was performed as described previously^42^. Briefly, proteins were obtained using radioimmunoprecipitation assay buffer, measured using Bradford assay, and used for immunoblotting. Proteins were detected using anti-LC3-I/II (sc-398822, Santa Cruz Biotechnology, Santa Cruz, CA, USA), anti-NF-κB1 (sc-8414, Santa Cruz Biotechnology), anti-β-actin (A5441, Sigma-Aldrich), and anti-Mouse IgG HRP-linked antibody (#7076S, Cell Signaling Technology, Beverly, MA, USA). Protein levels were visualized by chemiluminescence using an X-ray film (EA8EC, AGFA, Mortsel, Belgium). Protein fold-changes were quantified using densitometry.

### GC/MS of PKSZ-EO

Identification of compounds in PKSZ-EO was analyzed and identified by GC/MS at the National Instrumentation Center for Environmental Management (Seoul National University, Korea). Briefly, GC/MS was performed using TRACE 1310 GC analyzer and ISQ LT-MS (Thermo Fisher Scientific), fitted with a 60 m × 0.25 mm DB-5MS GC column (0.25 μm film thickness, 1 mL/min flow rate) (Agilent, Santa Clara, CA, USA). The oven temperature was programmed from 50 ℃ for 5 min, 50–65 ℃ at 10 ℃/min, 65–210 ℃ at 5 ℃/min, 210–310 ℃ at 20 ℃/min, and 310 ℃ for 10 min. The transfer line and ion source temperatures were 300 and 270 °C, respectively. Masses were acquired from m/z 35–550 at a rate of 0.2 scans/sec. Retention indices (RI) were identified using n-alkane standards (C7–C30). Metabolites were identified by comparing MS spectra and RI with NIST11 mass spectral library Ver. 2.0 g. The final identification was performed by comparing the retention time (RT) and MS spectra with those of commercially available standards. The chemical structure was illustrated using ChemDraw Ultra 12.0 (PerkinElmer, Waltham, MA, USA).

### Image processing and analysis

The contrast and brightness were optimized using Photoshop software ver. 2022 (Adobe Systems, San Jose, CA, USA) to properly display the representative images. The uncropped and unadjusted images is shown in supplementary materials. Densitometry was performed using ImageJ ver.1.5.3q (National Institutes of Health, Bethesda, MD, USA).

### Statistical analysis

All results are expressed as the mean ± standard deviation. Statistical analysis was performed using an unpaired two-tailed Student’s *t*-test or analysis of variance (ANOVA) with Tukey’s multiple comparison test using GraphPad PRISM. Statistical analysis used for each result is shown in the figure legend. p values < 0.05 were considered statistically significant.

## Electronic supplementary material

Below is the link to the electronic supplementary material.

Supplementary Material 1

Supplementary Material 2

## Data Availability

All data presented in this study are available within the manuscript and supplementary material.
