# Supplementary material for: Pinus koraiensis essential oil enhances glucose uptake and proliferation in SH-SY5Y neuroblastoma cells
Source: Sci Rep. 2024 Nov 4;14:26630. doi: 10.1038/s41598-024-78357-8 (PMC11535478; doi:10.1038/s41598-024-78357-8)
Supplement: Supplementary file 1 — Supplementary Material 1 [file 41598_2024_78357_MOESM1_ESM.docx]

**Abbreviations**

GLUT: glucose transporter · GD: glucose depletion · EO: essential oil · PKSZ: *Pinus koraiensis* Siebold & Zucc. · EO: essential oil
