# Supplementary material for: Pinus koraiensis essential oil enhances glucose uptake and proliferation in SH-SY5Y neuroblastoma cells
Source: Sci Rep. 2024 Nov 4;14:26630. doi: 10.1038/s41598-024-78357-8 (PMC11535478; doi:10.1038/s41598-024-78357-8)

**[Supplementary materials]**

***Pinus koraiensis* essential oil enhances glucose uptake and proliferation in SH-SY5Y neuroblastoma cells**

Hyungkuen Kim, Hwan Myung Lee, Sung-Jo Kim^*^

Department of Biotechnology, College of Life and Health Sciences, Hoseo University, Baebang, Asan 31499, South Korea

*** Corresponding author**

Sung-Jo Kim: E-mail: sungjo@hoseo.edu, Tel: +82-41-540-5571

**Contents**

**1. Supplementary table**

Table S1. Raw data for qRT-PCR in this study.

**2. Uncropped and unadjusted images**

**1. Supplementary table**

**Table S1. Raw data for qRT-PCR in this study.** mRNA fold changes normalized to RPL13A using the 2(^-ΔΔCt^) method

| **Target** | **Vehicle** | | | **PKSZ-EO** | | |
| --- | --- | --- | --- | --- | --- | --- |
|  | **#1** | **#2** | **#3** | **#1** | **#2** | **#3** |
| *SLC2A1* | 1.1093 | 1.0000 | 1.1961 | 0.4868 | 0.4280 | 0.3726 |
| *SLC2A2* | 3.0074 | 1.0000 | 0.9167 | 4.8449 | 4.2995 | 3.1738 |
| *SLC2A3* | 1.0885 | 1.0000 | 1.3036 | 1.9876 | 2.2134 | 2.3537 |
| *SLC2A4* | 1.4729 | 1.0000 | 1.1482 | 30.6961 | 24.2097 | 22.3448 |
| *LDHA* | 1.0008 | 1.0000 | 1.2428 | 0.8402 | 0.7636 | 0.7523 |
| *SLC16A1* | 1.5463 | 1.0000 | 1.4199 | 2.8839 | 2.5199 | 2.4849 |
| *SLC18A2* | 1.3629 | 1.0000 | 0.0141 | 10.6738 | 8.7260 | 2.9957 |
| *VAMP1* | 1.3551 | 1.0000 | 0.9975 | 2.2029 | 1.7621 | 2.0383 |
| *VAMP2* | 1.1294 | 1.0000 | 1.0096 | 1.1808 | 1.3761 | 1.3438 |
| *SYT1* | 0.9734 | 1.0000 | 0.8424 | 1.1836 | 1.1655 | 1.2428 |
| *PTGS2* | 1.0253 | 1.0000 | 1.1472 | 0.5043 | 0.3002 | 0.5206 |
| *IL10* | 3.9461 | 1.0000 | 0.3127 | 6.7328 | 6.6536 | 5.7184 |
| *PNPLA2* | 0.9137 | 1.0000 | 1.1448 | 2.2298 | 1.9117 | 1.7315 |

**2. Uncropped and unadjusted images**


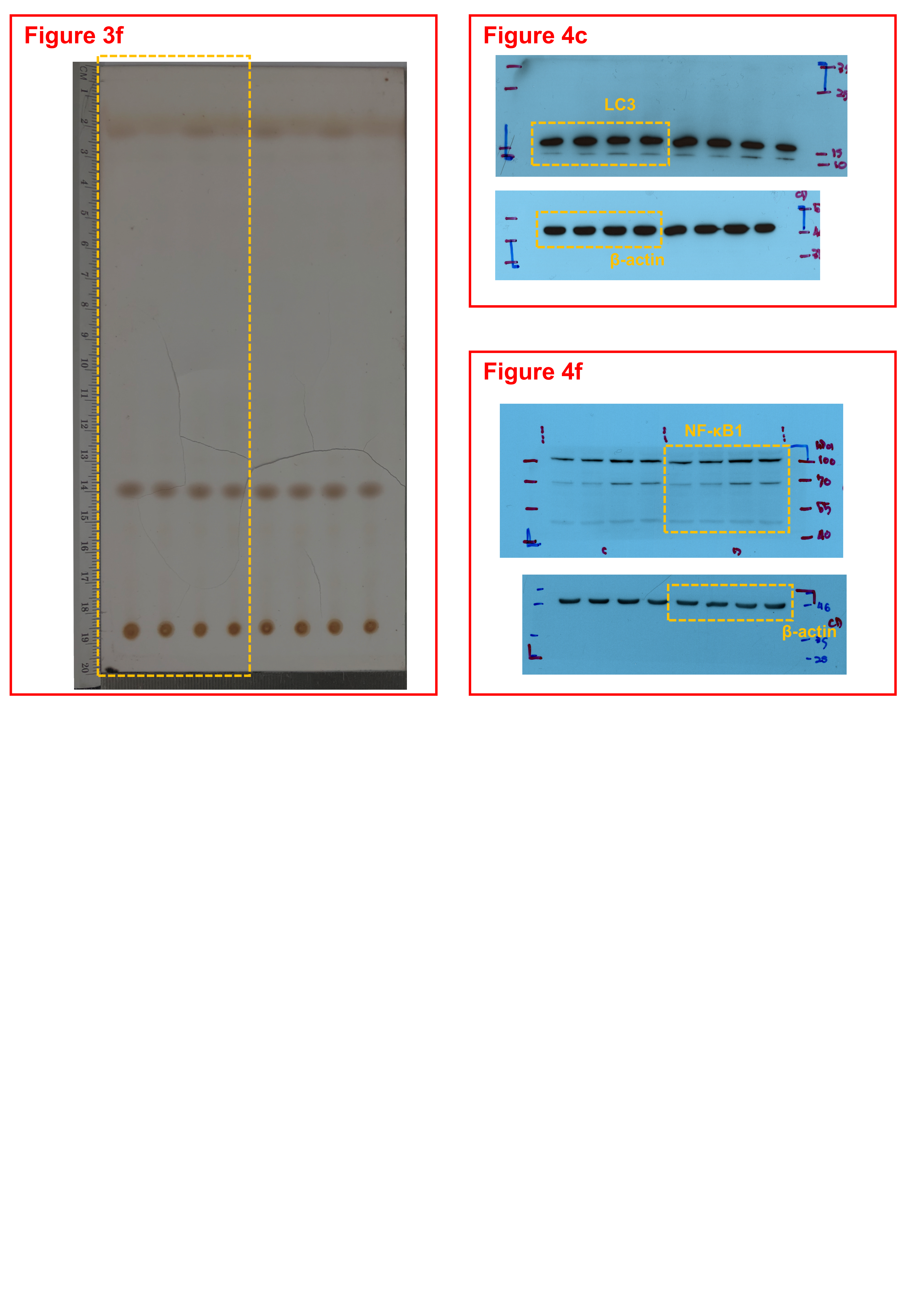

Supplement: Supplementary file 2 — Supplementary Material 2 [file 41598_2024_78357_MOESM2_ESM.docx]
